# Extending Working Life: Which Competencies are Crucial in Near-Retirement Age?

**DOI:** 10.1007/s10804-017-9274-9

**Published:** 2017-10-17

**Authors:** Justyna Wiktorowicz

**Affiliations:** Department of Economic and Social Statistics, Faculty of Economics and Sociology, University of Lodz, Rewolucji Street 41, 90-214 Lodz, Poland

**Keywords:** Active ageing, Extending working life, Competencies, Multivariate statistics, Poland

## Abstract

Nowadays, one of the most important economic and social phenomena is population ageing. Due to the low activity rate of older people, one of the most important challenges is to take various actions involving active ageing, which is supposed to extending working life, and along with it—improve the competencies of older people. The aim of this paper is to evaluate the relevance of different competencies for extending working life, with limiting the analysis for Poland. The paper also assesses the competencies of mature Polish people (aged 50+, but still in working age). In the statistical analysis, I used logistic regression, as well as descriptive statistics and appropriate statistical tests. The results show that among the actions aimed at extending working life, the most important are those related to lifelong learning, targeted at improving the competencies of the older generation. The competencies (both soft and hard) of people aged 50+ are more important than their formal education.

## Introduction

Nowadays, one of the most important economic and social phenomenon is population ageing. It is usually perceived as a threat to public finances, the healthcare system, the social protection system, and the stable functioning of business entities. However, it should be noted that more and more people no longer perceive the issue in these terms. The ‘silver economy’, age management, and lifelong learning are concepts that are taking root in the public awareness, and they are all orientated towards making the best use of the potential of people nearing retirement age or retired (they form part of the “life cycle” concept). They also cater to new social needs that accompany, on one hand, the increasing social participation of people over 50 and, on the other, a marked improvement in their quality of life. These issues are a part of the active ageing concept, which is becoming a dominating paradigm in economic and social policy all over the world and concentrates on the “economisation” of extending working life (OECD 1998, 2006). In references and official EU documents, active ageing is usually defined as the process of optimising possibilities in the range of health, participation, and safety, which is supposed to improve the quality of life for people as they are ageing (WHO 2002). The word “active” applies not only to physical or economic activity, but also to constant participation in the economic, cultural, spiritual, and civic life of societies (EC 2012). Of critical importance are the different actions involving active ageing aimed at extending working life.

The crucial assumption of the active ageing concept is the idea of the necessity to affect as long as possible the maintenance of an individual’s productivity. Productivity of older people could be understood, in the wider sense, as social productivity defined as any activity that produces goods and services, whether paid or not, including activity such as housework, childcare, voluntary work, and help to family and friends. From the labour market and workplaces perspective, productivity is understood in limited sense as economic productivity. Of critical importance are in this context the different actions aimed at improving human capital (including competencies improvement) of older people, which is the main indicator of individual productivity. In this paper, the analysis is conducted from the workforce potential perspective, which determines its restricted approach, with taking into account only the economic productivity, intentionally omitting wider sense (i.e. social productivity).

In Poland, as in other countries of the European Union, the situation on the labour market is increasingly determined by the deepening processes of population ageing. Currently, Poland is a relatively young EU Member State as far as demography is concerned—the age median and the economic dependency ratios are below the EU average. But the current economic activity of people at near-retirement age is low. According to Eurostat, in 2016 the activity rate of the Polish people aged 50–64 was about 57.3% (and only 49.5% for women), and the employment rate was 54.7% (for women—only 47.5%). In EU-28, these indicators were on the levels, respectively, of 67.8 and 61.1% for women (activity rate) and 63.4 and 57.3% for women (employment rate). Using Eurostat data, we could also note that only 5.6% of people aged 65+ (9.3% of people aged 65–74) are employed (in Poland, respectively—4.9 and 7.6), but there are such countries as Sweden, the UK, and Ireland, where the employment rates for people aged 65–74 are about 15% and Estonia with the indicator of 25.3%. In UE-28, employment rate for women aged 65+ is only 3.7% (in Poland—2.7%, but in) and for women aged 65–74 it is 6.6% (in Poland—only 4.4). Additionally, although the age difference between men’s and women’s statutory retirement age (about five years), the effective retirement age of men and women was in Poland almost the same—about 60 (Przejście … 2013). In 2013, about 14% of the total number of retired persons were people below the statutory retirement age (Emerytury … 2014). These facts imply the need to intensify actions aimed at extending the working life of Polish people. In accordance with the act applicable from 1 January 2013, the retirement age for men and women is to be gradually raised to 67 years (women born after 30 September 1973 and men born after 30 September 1953 will acquire the right to retire at age 67). The pathways to early retirement were also restricted in Poland. On the other hand, with the help of the European Social Fund, actions are being instituted connected with the surroundings of the work environment. These actions focus on extending the working life and making better use of the potential of people aged 50+ and are regulated by a government programme called “The solidarity of generations. Actions for increasing the economic activity of people aged 50+” (2008). The planned intervention measures targeted at this group comprise those directed at (1) the unemployed, which are included in active labour market policy (ALMP), and (2) the employed, in order to enhance or extend their working life. The main factor is equalisation of opportunities for older workers in the labour market, which is directly related to expanding the qualifications of persons belonging to this group (Urbaniak and Wiktorowicz 2014). Unfortunately, the participation of people in near-retirement age in education and training is low. This raises the following questions: Maybe the trainings are not required? Maybe the competencies of older Poles are already sufficient? What is the role of the competencies of mature people in the context of the choice between work and retirement?

The aim of this paper is to evaluate the relevance of different competencies for extending working life understating as maintaining older workers in official employment. It also assesses the competencies of mature Poles (aged 50+).

## Determinants of Extending Working Life—Literature Review

The policy of extending working life has been a significant outcome of the debate concerning the economic sustainability of ageing populations, and reflects in large measure the pressures identified in the preceding section (Maltby 2011; Weyman et al. 2012). The aim is to reverse the trend—characteristic of the 1980s and 1990s (in Poland observed in the period 1990–2005)—whereby older workers left work at earlier ages, and where early retirement came to be accepted as a normal event (Marshall et al. 2001).

In line with previous studies, factors for the extension of working life can be located on both the macro- and meso-, but especially on the microeconomic level. Among the most important the following characteristics should be listed:

### Factors at the Macro- and Mesoeconomic Levels

- Difficult situation on the labour market or the instability of the labour market (people decide to retire because of the lack of jobs in their place of residence, especially in little towns far away from big cities. Moreover, they are constantly afraid of losing their jobs; in this situation, retirement seems to be something stable and it does not exclude one from the labour market) (Giza-Poleszczuk et al. 2008).
- Incentives for retirement that are present in a social security system: the instability of law (people decide to retire because they are afraid that the system concerning retirement will change adversely); the availability of jobs on the “black market”; the relationship between social benefits and salaries (especially in the case of less-paid and unskilled jobs); and the possibility of combining retirement with work (Giza-Poleszczuk et al. 2008).
- Employers’ negative attitude towards hiring older employees and keeping them employed, especially when there is a growing disproportion between decreasing productivity and the salaries of older people (Gurria 2008; Kołodziejczyk-Olczak 2014; Munnel and Sass 2008; Perek-Białas et al. 2010; Wiśniewski 2009).

### Determinants on the Individual Level

- Psychological barriers: a lack of motivation, low work ethic, low self-esteem, and lack of good work habits in the case of persons who have been unemployed for a long period of time (Determinanty… 2010; Claes and Heymans 2008; Groot and Brink 1999).
- Demographic features, especially sex (women are under pressure to stay unemployed when they do the housekeeping or take care of the home, children, and sick people, which encourages their earlier retirement); education (better-educated people are more likely to be employed, regardless of other limitations); place of residence (which is related to the availability of job offers); and age (Woszczyk 2011; Silcock 2012; Contreras et al. 2013; Kryńska et al. 2013; Naegele and Bauknecht 2013; Humphrey et al. 2003; Khan et al. 2014; Wiktorowicz 2014).
- Health (which deteriorates with age), and physical and mental exhaustion (Rzechowska 2010; Maitland 2010; Kryńska et al. 2013; Naegele and Bauknecht 2013; Phillipson and Smith 2005).
- Professional position—longer economic activity is planned by managers, specialists, self-employed people, people employed on the basis of a permanent or long-term contract, and people who are appreciated by co-workers and supervisors (Silcock 2012; Contreras et al. 2013; Summary … 2012; Kryńska et al. 2013).
- Difficult family situation (disease or disability of a family member) or positive aspects of a family life. People deepen and discover new aspects of marriage (therefore people tend to retire together with their spouses), and in addition elderly persons become grandparents (Rzechowska 2010; Kotowska and Wóycicka 2008; Kryńska et al. 2013; Scherger et al. 2012; Whiting 2005; Smeaton and McKay 2003; Davis et al. 1992; Wiktorowicz 2014).
- Desire to fulfil oneself in aspects of life not related to work (Kryńska et al. 2013).

Outdated professional qualifications of older people are also indicated as an important factor which causes persons to cease their economic activity and retire, being at the same time a barrier to extending one’s working life (Gurria 2008; Sinclair et al. 2013; McNair et al. 2004). As Reeve et al. (2012) indicate, competencies change with age. For older workers, social skills are an especially relevant competence (Hennekam 2015). It should be noted that older workers are often thought to have better interpersonal skills (Rosen and Jerdee 1976) than younger workers (but for younger workers their value is higher—Murakami et al. 2008). Cunningham and Sweet (2009) found that social skills are frequently used in order to adapt successfully during one’s career, and social skills (along with integrity and motivation) have a positive relationship to job satisfaction and extrinsic career success (Hennekam 2015). On the other hand, Kroll (2003) asserted that in view of the accelerated technical and organisational changes, older workers are confronted with the risk of “de-qualification”, i.e. the downgrading of their acquired expertise or personal competencies. According to widely formulated gerontological theory, older people do not follow new technologies and forms of communication, and when put in a situation of mounting pressure, older people tend to withdraw (Szukalski 2008). Thus, the main problem in the context of further employment of older workers is the “devaluation” of competencies rather than ageing itself. Computer skills still seem to be a gap in older persons’ development (Hashin and Wok 2011). Summarising, it should be pointed out that generally the role of competencies in the context of extending one’s working life or career is rather rarely investigated (Bridgstock 2011; Kong et al. 2012; Kuijpers and Scheerens 2006; Kautonen et al. 2012).

## Data and Methods

### Data

An empirical analysis was performed on the basis of the data provided by Bilans Kapitału Ludzkiego (BKL, The Study of Human Capital) for 2013.^1^ This multi-module study aimed at a comprehensive diagnosis of human capital in Poland and included, among others, the population in working age, i.e. women aged 18–59 and men aged 18–64. The study is representative for the whole population of Poles in working age and for a cross section of sexes, age groups, and voivodeships (i.e. regions—NUTS2). It was conducted in each edition on a sample of about 17,600 people.

Because the main topic of this paper is the analysis of the competencies of Poles in the context of their decision to retire, the population was restricted to people aged 50–59/64 (further called 50+). The upper limit of the researched population was the limit of their working age. The lower limit was established according to the meaning of “older age” as commonly understood in Poland (and also in other EU countries) in view of the labour market situation. This, in turn, is based on the legal identification of older participants in the labour market as a disadvantaged group.^2^ In the BKL 2013, people aged 50–59/64 constituted around 28% of the sample (*n* = 4999.^3^)

### Measurement of Competencies

The methods for measuring competencies may be as varied as the definitions used to describe them. In the light of one of the first definitions (Boyatzis 1982), competence is the potential, existing in a human, to attain and/or maintain such a behaviour that helps satisfy the requirements of a given post within an organisational environment, which in turn provides the desired results. Armstrong (2000) defined competencies as a potential which helps achieve defined (desired) results, and Bjornavold and Tissot (2000) as corroborated personal skills to use know-how, qualifications, and knowledge to meet the current and potential professional challenges. A similar definition was used by Filipowicz (2004)—competencies are the predispositions in the area of knowledge, skills, and attitudes that allow an individual to conduct professional tasks at an appropriate level. In the Study of Human Capital, competencies are defined similar to the definition of Filipowicz—as knowledge, skills, and attitudes associated with the performance of specific actions, independent of the mode in which they were acquired, and whether they have been corroborated with a validation procedure (Górniak et al. 2011).

The measurement of competencies is performed in various ways. On the organisational level, the most important are observational scales and questionnaires, competency tests, behavioural surveys, and an Assessment & Development Center (Filipowicz 2014). Of course not all of these methods may be put to use in social research aiming at the diagnosis of all people in productive age. Therefore in BKL, competencies were measured through self-assessment of a respondent’s competency levels (especially because of the idea to perform a holistic diagnosis of supply and demand for competencies on the labour market).^4^ The respondents were given the following instructions: “different types of work require different skills and talents. Yet frequently it is so that in one or two areas, our capacity is rather high, while in others it is far lower. Moreover, everyone has a certain image of the work that he would like to perform. On the one hand, sometimes we can do something very well, but we do not want to do work related to it. On the other hand, we may want very much to perform certain works, but as yet do not have sufficient skills. Now I’m going to read a list of different skills to you. For each of them, I will ask you to assess the level of your skill in this area on a 5-point scale, where 1 denotes a very low level; 2—basic; 3—medium; 4—high; and 5—very high”. The list of competencies used in the BKL 2013 is presented in Table 1.

**Table 1:** Competencies classes used in BKL’2013 study. (Source: BKL 2014, 2011)

No.	Competence	Dimension of the behaviour	Subdimension of the behaviour
K1	Cognitive	Finding and analysing information and drawing conclusions	Quick summarisation of large volumes of text
			Logical thinking, analysis of facts
			Continuous learning of new things
K2	Technical	Operating, assembling, and repairing devices	–
K3	Mathematical	Performing calculations	Performing simple calculations
			Performing advanced mathematical computations
K4	Computer	Handling computers and using the internet	Basic knowledge of MS Office-type package
			Knowledge of specialist software, ability to write applications and author websites
K5	Artistic	Artistic and creative skills	–
K6	Physical	Physical fitness	–
K7	Self-organisation	Self-organisation of work and showing initiative, timeliness	Independent making of decisions
			Entrepreneurship and showing initiative
			Creativity (being innovative, inventing new solutions)
			Resilience to stress
			Timely completion of planned actions
K8	Interpersonal	Contacts with other people	Cooperation within the group
			Ease in establishing contacts with colleagues and/or clients
			Being communicative and sharing ideas clearly
			Solving conflicts between people
K9	Office	Organising and running office work	–
K10	Managerial	Managerial skills and organisation of work	Coordination of work of other staff
			Disciplining other staff—taking them to task
K11	Availability	Availability	Readiness to travel frequently
			Flexible working hours (no fixed slots)
K12	Language	Fluent command of Polish spoken and written	–

When identifying the importance of assessing the competencies of Polish people aged 50+ for their economic activity, an attempt was made to aggregate the traits listed in Table 1. This task was completed in two ways (all are counted as the sum of the suitable subvariables, each time omitting the K12 competency due to other reasons for its inclusion in BKL):

1. Variant A—synthetic variables were created to provide quantitative measurement of cognitive (*W1*_*A*_), mathematical (*W3*_*A*_), computer (*W4*_*A*_), self-organisation (*W7*_*A*_), interpersonal (*W8*_*A*_), managerial (*W10*_*A*_), and availability (*W11*_*A*_) competencies; other competencies (*K2, K5, K6*, and *K9*) were left in their original form;
2. Variant B—synthetic variables measured competencies in general (*W*_*B*_), soft competencies (*WM*_*B*_, including interpersonal, self-organisation, cognitive, availability, managerial, artistic, and creative competencies), and hard competencies (*WT*_*B*_: technical, physical, office, mathematical, and computer competencies).

### Measurement of the Situation on the Labour Market and its Conditions

BKL is at the same time the best source of information about Polish people’s competencies. However, in the context of assessment of the economic activity of the older generation, the information resources of this study are not very large. The situation on the labour market can be assessed on the basis of the answer to the following question: “How would you assess your current job situation?”, since there an “I’m retired” option is included among the answers.

The evaluation of the relationship between economic activity and the competencies of people aged 50+ was based on the current state of both features at the time of the study. Therefore, the analysis does not allow for drawing direct cause–effect relationships between the respondents’ competencies and their decision to retire. To do that, their competencies at the moment of retiring should be considered, but this is impossible due to the nature of the study (it is not a typical panel study), and the lack of data about the year of retiring.^5^ Nevertheless, considering that the study includes men and women still in the productive age, the results may form the basis for conclusions about the relationship between competencies and termination of one’s economic activity due to the retirement of people aged 50+. The analysis comprises (1) a comparison of the competencies of people aged 50+ in both the economically active and inactive (due to retirement) groups and (2) constructing and verifying an analytical model describing the risk of terminating one’s economic activity due to retirement.

### Statistical Analysis

The analysis was conducted with the application of basic descriptive statistics, as well as the Student’s *t* test for independent samples, the Mann–Whitney test, the *χ* ^2^ test of independence, and the tau Goodman–Kruskal coefficient. Additionally, using logistic regression, the probability of retirement was estimated. The termination of economic activity due to retirement (output variable in the model) was measured as a dummy variable (*Ret*), taking on the value of 1 if the respondent described their job situation at the time of the study as “retired”, and 0 if they were economically active (which excluded people economically inactive because of reasons other than retirement). Explanatory variables were the following:

- competencies measured according to variants A (model 1.A) and B (model 1.B) and
- sex, place of residence (the region of Poland and size class of the location), level of education, years of economic activity, age, health (answer to the question: “Do any long-term illnesses, disabilities, other health conditions or mental health conditions hinder your performance of everyday tasks?”), marital status, spouse’s retirement, and competencies measured according to, as appropriate, variants A (model 2.A) and B (model 2.B).

In all calculations, the standard level of significance (*α* = 0.05) was adopted. The calculation was made in SPSS 22.0.

## Assessment of the Competencies of Polish People Aged 50+

In general, people aged 50+ assess their soft competencies as better than their hard competencies. The highest marks were given to interpersonal competencies (K8)—all skills from this group were assessed similarly: almost 90% people aged 50+ consider them at least “average”, and almost 2/3 as at least “good” (the median of the marks is 4, and the mean is about 3.7). Self-organisation competencies were marked highly, too (especially timely completion of planned actions and independent making of decisions—where the median was also four and the mean was about 3.5). People aged 50+ also highly assessed their availability, cognitive and managerial competencies, especially “flexible work hours” and logical thinking, analysis of facts, and—in contrast with common stereotypes—continuous learning of new things. The results of the Study of Human Capital confirm that the computer competencies of older Poles (still in the working age) remain low—less than half of them consider them at best average, and only 1/5 mark them as good or very good. The lowest mark (median only 1) refers to the knowledge of specialised programmes and the ability to write applications and author websites, although their knowledge of MS Office is also not very good (median 2). Considering it as one of the basic contemporary requirements of new employees, it places people aged 50+ at a disadvantage when compared with younger people on the labour market.

Taking into consideration synthetic indicators of competencies, it should be noted their high or at least acceptable reliability (Cronbach’s alpha *α* > 0.6—Table 2). This means that although some of them are created based on short scales (W3_A_, W4_A_, W3_A_, and W11_A_ are based on only two partial indicators), they allow for a reliable description of the competencies of Polish people aged 50+. Importantly, the constructed indicators are measured on a quantitative scale.

**Table 2:** Measurement of the reliability of synthetic indicators of competencies. (Source: As in Fig. 1)

Measure	Variant A							Variant B		
	W1_A_	W3_A_	W4_A_	W7_A_	W8_A_	W10_A_	W11_A_	W_B_	WM_B_	WT_B_
Range of variability	3–15	2–10	2–10	5–25	4–20	2–10	2–10	24–120	17–85	7–35
α-C	0.867	0.691	0.775	0.883	0.879	0.898	0.792	0.946	0.940	0.801
Mean	9.23	5.62	3.74	16.53	14.24	5.69	6.06	71.95	54.08	17.89
Median	9.00	6.00	3.00	17.00	15.00	6.00	6.00	72.00	55.00	17.00
Standard deviation	2.69	1.96	2.07	4.39	3.48	2.31	2.24	17.85	13.28	5.50
Skewness	− 0.043	0.238	1.104	− 0.314	− 0.548	0.001	− 0.133	− 0.110	− 0.257	0.410
Kurtosis	− 0.324	− 0.553	0.349	− 0.210	0.202	− 0.923	− 0.768	−0.204	− 0.109	− 0.286

**Fig. 1:**
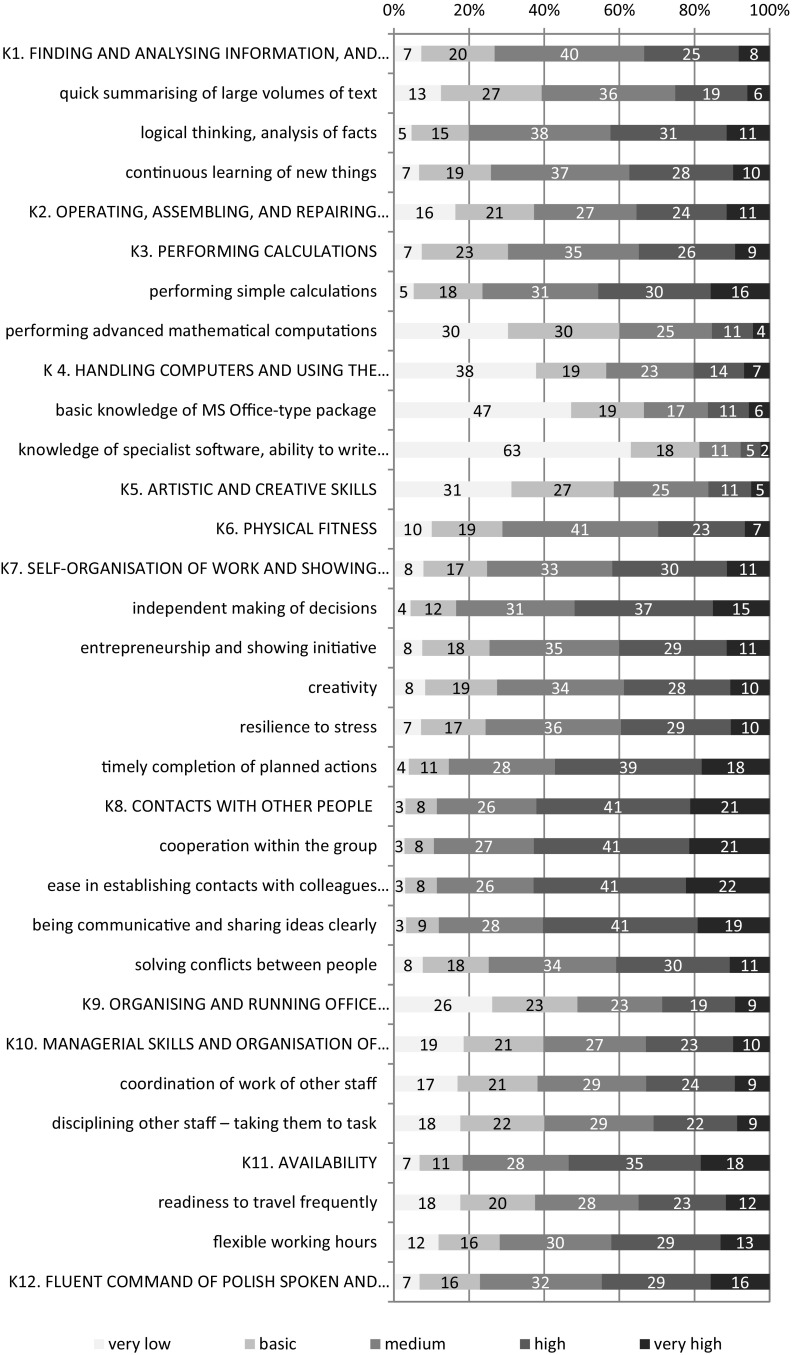
Self-assessment of the competencies of Polish people aged 50+ (in %). [Source: Own calculations based on individual BKL 2013 data (http://bkl.parp.gov.pl/dane)]

An analysis of the descriptive statistics confirms a quite high level in most of the examined competencies of Polish people aged 50+, with the exception of computer competencies (W4_A_), for which the median is only 3 points (mean 3.74), while the maximum value for this feature is 10 points. At the same time, the variability of computer competencies among the studied population is high (standard deviation approx. 2 points). Besides, it is the only competency with a high right-skewed distribution (among people aged 50+ some have unusually high computer competencies). For the other indicators, their statistics point to a quite high competency level, rather low variation, rather even distribution in relation to the mean, and a distribution similar to the appropriate normal distribution. Soft competencies are, generally speaking, assessed higher than the hard ones—the mean for the first group (WM_B_) is about 54 points (with a maximum of 85 points), while for the second group (WT_B_) it is about 18 points with a maximum of 35 points.

## Competencies and Economic Activity

**Fig. 2:**
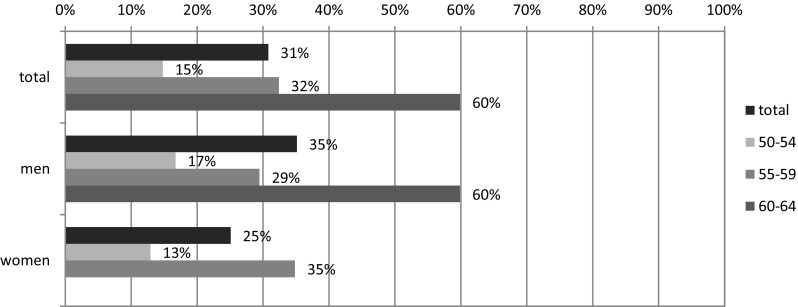
The scope of retirement among people aged 50 + according to sex and age groups (%). (Source: As in Fig. 1)

In light of the BKL research, almost a third of people aged 50–59/64 are retired (Fig. 2). Of course, this percentage increases in the subsequent 5-year age groups. In the five years preceding the limit of working age, six in ten men and more than one-third of women are retired, while 13% of women and 17% of men declared their termination of economic activity due to retirement already in the age of 50–54.

Retirement prior to normal retirement age is significantly related (in the statistical sense) with the competencies of people aged 50+—both the hard and soft ones (in Student’s *t* test, p < 0.001). In analysing individual competencies, significantly higher levels were diagnosed in the population of professionally active Poles aged 50–59/64 than the retired ones (lack of significant differences appeared only in the case of technical competencies—*p* = 0.853).^6^ The relatively largest differences (to the pensioners’ disadvantage) appeared with computer competencies as well as availability and physical fitness. When analysing individual competencies at the second stage of their classification, the large role of knowledge of MS Office, readiness to travel frequently, and flexible work hours should be noted, as well as the continuous learning of new things and creativity. Further down the list were entrepreneurship, showing initiative, independent making of decisions, timely completion of planned actions, ease in establishing contacts, and resilience to stress. Analysing the value of Goodman and Kruskal’s coefficient tau (*τ*), which allows to measure the strength of the cause–effect correlation between the qualitative variables, it can be determined that the factors which most affect the decision to retire most are physical fitness (*τ* = 0.043) and availability (*τ* = 0.043). This pertains more to men (respectively, *τ* = 0.050 and *τ* = 0.040) than women (*τ* = 0.034 and *τ* = 0.035). In the case of women, managerial skills, creativity, and a basic knowledge of MS Office play a bigger role compared with men.

The probability assessment of terminating economic activity due to retirement was performed using logistic regression.^7^

The results of the estimation given in Table 3 show that low competencies are a significant but not sole factor in leaving the workplace due to retirement—in exploring their influence on retirement, the obtained models had very low prognostic values (as evidenced especially by the low value of the Nagelkerke coefficient) and almost nonexistent classification properties. Among all competencies, six are significant for terminating economic activity due to retirement—assuming *ceteris paribus* (model 1A, Table 3):

**Table 3:** Results of estimation of the logistic regression model (version A). (Source: As in Fig. 1)

Independent variables	Model 1A		Model 2A	
	OR	*p*	OR	*p*
Computer	0.865	< 0.001*	0.892	0.001*
Self-organisation	0.968	0.040*	0.947	0.005*
Managerial	1.107	< 0.001*	1.109	0.002*
Availability	0.827	< 0.001*	0.844	< 0.001*
Technical		0.002*		
Basic	1.207	0.248		
Medium	1.580	0.003		
High	1.488	0.013		
Very high	1.989	< 0.001		
Artistic		< 0.001*		0.001*
Basic			1.331	0.046
Medium			1.336	0.061
High			1.556	0.037
Very high			3.194	< 0.001
Physical fitness		< 0.001*		0.011*
Basic	1.060	0.769	1.009	0.973
Medium	0.568	0.003	0.642	0.069
High	0.437	< 0.001	0.613	0.068
Very high	0.309	< 0.001	0.474	0.035
Region				< 0.001*
South			2.219	< 0.001
East			0.988	0.949
Northwest			0.914	0.642
Southwest			1.056	0.797
North			1.231	0.294
Place of residence				0.005*
Rural area			1.975	0.001
Urban area, below 20,000 inhabitants			2.151	0.001
Urban area, 20,000–49,999 inhabitants			2.295	< 0.001
Urban area, 50,000–99,999 inhabitants			2.005	0.005
Urban area, 100,000–499,999 inhabitants			1.518	0.073
Marital status				< 0.001*
Divorced/separated			1.422	0.110
Widowed			2.072	< 0.001
Single			0.739	0.115
Age (in years)			1.340	< 0.001*
Spouse’s retirement			3.078	< 0.001*
Health			1.361	0.026*
Constant	0.126	< 0.001*	0.000	< 0.001*
p in Hosmer–Lemeshow test	0.280		0.172	
*R* ^2^ Nagelkerke	0.108		0.400	
Quality of classification (for retirees)	29.9%		64.4%	
Observations	3686		3686	

*Reference group* region—central; place of residence—urban area, over 500,000 inhabitants; spouse’s retirement—wife/husband retired; marital status—people in relationships (formal or non-formal); health—people, whose conditions do not hinder their normal functioning; technical and artistic competencies, physical fitness—very poor

*OR* odds ratio, *p* probability in Wald test

*Relation statistically significant (*α* = 0.05)

- Computer and availability competencies—an increase in their general assessment by 1 point decreases the risk of terminating economic activity due to retirement by 13–17% on average.
- Self-organisation competencies—an increase in their general assessment by 1 point decreases the risk of terminating economic activity due to retirement by 3% on average.
- Technical competencies—the higher they are, the higher the risk of terminating economic activity due to retirement; compared to people 50+ with very poor technical competencies, for people with average or higher competencies this probability is 1.6–2 times higher.
- Physical fitness—compared to people with poor physical fitness, the risk of terminating economic activity due to retirement is almost 1% higher for people with basic physical fitness; for people with average physical fitness, the risk of terminating economic activity due to retirement decreases by about 45%; for people with high physical fitness, it decreases by about 55%; and for people very fit physically it decreases by about 70%.
- Managerial competencies—an increase in their general assessment by 1 point increases the probability of terminating economic activity due to retirement about 1.1 times on average.

The results obtained for technical and managerial competencies are rather surprising—it would seem that all competencies would promote economic activity, i.e. the higher their level, the longer the activity. The results for technical competencies are easy to explain by the relation with the kind of work performed—people with higher levels work more frequently in jobs requiring physical effort, and therefore the probability of their retirement is higher. Besides, a significant positive relationship between these competencies and age was observed—in the group aged 50+, higher competencies are prevalent among people in the later phase of their working age, where the percentage of those retired is higher compared with the other groups. The results with respect to managerial competencies remain surprising, especially because the percentage of the retired is, generally speaking, higher for people who assess their skills in coordinating the work of other staff and disciplining other staff as weak.

Taking into consideration the summarily measured hard and soft competencies (variables *WM*_*B*_ and *WT*_*B*_), it should be noted that if only these variables were included in the logistic regression model did both of them have a significant influence—hard competencies higher by 1 point lessen the risk of terminating economic activity due to retirement on average by 3.4%, and soft competencies on average by 2.2% (model 1B.1, Table 4). The coexistence of a high level of hard and soft competencies has the especially high importance (the effect of the interaction of these two factors is statistically significant), reducing the risk of terminating economic activity due to retirement on average by 0.1% (model 1B.2, Table 4).

**Table 4:** Results of estimation of the logistic regression model (version B). (Source: As in Fig. 1)

Independent variables	Model 1B.1		Model 1B.2		Model 2B.1		Model 2B.2	
	OR	*p*	OR	*p*	OR	*p*	OR	*p*
Soft competencies (WM_B_)	0.978	< 0.001*			0.973	< 0.001*		
Hard competencies (WT_B_)	0.966	0.006*						
Interaction WM_B_ and WT_B_			0.999	< 0.001*			0.999	< 0.001*
Region						< 0.001*		< 0.001*
South					2.141	< 0.001	2.148	< 0.001*
East					1.057	0.766	1.043	0.822
Northwest					0.931	0.704	0.911	0.619
Southwest					1.113	0.603	1.125	0.569
North					1.208	0.331	1.190	0.369
Place of residence						0.005*		0.010*
Rural area					1.938	0.002	1.876	0.003
Urban area, below 20,000 inhabitants					1.965	0.004	1.946	0.004
Urban area, 20,000–49,999 inhabitants					2.227	0.001	2.204	0.001
Urban area, 50,000–99,999 inhabitants					2.086	0.003	2.027	0.004
Urban area, 100,000–499,999 inhabitants					1.485	0.084	1.498	0.077
Marital status						< 0.001*		0.002*
Divorced/separated					1.198	0.198	1.303	0.224
Widowed					1.862	< 0.001*	1.849	0.001
Single					0.800	0.233	0.814	0.269
Age (in years)					1.362	< 0.001*	1.365	< 0.001*
Spouse’s retirement					3.100	< 0.001*	3.089	< 0.001*
Health					1.635	< 0.001*	1.585	< 0.001*
Sex					1.466	0.002*	1.468	< 0.002*
Constant	1.434	0.078	0.552	< 0.001*	0.000	< 0.001*	0.000	< 0.001*
p in Hosmer–Lemeshow test	0.086		0.086		< 0.001*		0.056	
R^2^ Nagelkerke	0.041		0.040		0.374		0.375	
Quality of classification (for retirees)	6.3%		1.2%		63.6%		63.7%	
Observations	3683		3683		3686		3686	

*OR* odds ratio, *p* probability in Wald test

*Relation statistically significant (*α* = 0.05)

As mentioned earlier, competencies are just one of the factors, but not the only one, in the termination of economic activity due to retirement among people aged 50–59/64. The inclusion of other features of this population, regardless of the version of the model, confirms on the one hand the importance of competencies, but on the other it points to the significance of the family situation of people aged 50+ (including marital status and economic activity of the spouse), health, age, sex, and place of residence (both the region and the size of the residential area). What may be surprising, the influence of the level of education turned out to be insignificant compared to other factors. Actually, the skills of people aged 50+ are more important than their formal education (see model 2A, Table 3 and models 2B.1 and 2B.2, Table 4). In turn, the significance of sex shows up only in models dividing the competencies into hard and soft ones (models 2B.1 and 2B.2).

Interpretation of the odds ratio for socioeconomic futures (excluding competencies) is analogous in all models, which confirms that the method of measurement of competencies does not affect the assessment. Assuming *ceteris paribus* after turning 50 years of age:

- With each following year of life after 50 the risk of terminating economic activity due to retirement grows on average 1.4 times;
- People with health conditions (i.e. whose conditions hinder their normal functioning) are on average about 1.6 times more prone to terminate economic activity due to retirement before reaching the general retirement age than people without these problems;
- Comparing people with various marital statuses, it was observed that the risk of terminating economic activity due to retirement is definitely the lowest for single persons (about 20% lower than the reference group, i.e. people in relationships); in turn for those divorced this risk is on average 1.2 times higher, and for the widowed it is about 1.8 times higher;
- People whose spouse is retired are over three times more likely to terminate economic activity due to retirement;
- Comparing Poles aged 50+ according to regions, it was observed that in relation to the central region (the Mazovia and Lodzkie voivodeships), only in the northwest region (the Lubuskie, Wielkopolska, and Zachodniopomorskie voivodeships) is the probability of retirement lower (by about 7%); in the remaining regions, the probability of retirement is higher, with the highest (over twice that of the central region) in the eastern regions (Lubelskie, Podlaskie, Podkarpackie, Świętokrzyskie), which are among the poorest in the EU;
- Inhabitants of smaller towns decide to retire before reaching the normal retirement age more frequently than inhabitants of the biggest cities; in particular, the inhabitants of medium-sized and large cities (50,000–499,000) made this decision almost twice less often than those in the biggest cities.

The prognostic and classification features of the models which included socioeconomic futures are much better than models that included only competencies. The quality of classification is over 60%, and the Nagelkerke coefficient is about 0.4. Nevertheless, the obtained results confirm that, apart from those described in the study other factors—both individual and macro- and mesoeconomic—are also of importance. These models do not take into account all the determinants of terminating economic activity due to retirement mentioned earlier (point 2), but, in accordance with the aim of this paper, do allow for an assessment of the role of competencies in economic activity.

## Conclusions

Stimulating the economic activity of the older generation is one of the most important tasks of contemporary social policy. The ageing of societies, with the simultaneous increase in average life spans and improvement in the quality of life (including for elderly persons), increases the focus on actions aimed at extending people’s working life and delaying the decision to retire. Compared with other EU members, Poland is one of the countries with the lowest employment indicators for older age groups, which makes such actions even more necessary.

Among the actions aimed at extending people’s working life, the most important ones are those related to lifelong learning, targeted at improving the competencies of the older generation. The research results presented in this paper confirm the need for such actions, although not all competencies stimulate longer economic activity. Those that do definitely include higher availability, computer and self-organisation competencies, as well as better physical fitness. Among people aged 50–59/64, the probability of retirement is higher for people with better technical competencies. Apart from competencies, health, marital status, place of residence (size and region), and, naturally, age are also significant factors. The wish to retire together with one’s spouse is clearly visible (if the spouse is retired, the risk of terminating economic activity due to retirement increases threefold, assuming all other features are stable). It should be noted that, assuming that all demographic and statistical features are on the same level for all units of the study, the influence of competencies remains statistically significant. The fact that both hard and soft competencies, and especially their coexistence, are significant, is also important. The results clearly confirm that the current assumptions of social policy in Poland should be maintained. Among the individual competencies, basic computer competencies (especially the knowledge of MS Office) should in particular be further developed. Entrepreneurship and resistance to stress should also be stimulated. Actions related to preventive health care and job ergonomics promoting better physical fitness among people 50+ are also essential. These aims are definitely easier to achieve in people more open to learn new things and more creative. Additionally, such people decide less often to retire before reaching the general retirement age. Actions increasing the geographical mobility of people 50+ and facilitating an open attitude towards flexible forms of labour organisation are also important. It should be noted that the assessment of the competencies of Polish people remains at a similar level in all editions of the BKL. The conclusions drawn so far may thus be expanded to include periods of time other than the time of the study. On the other hand, it should be stressed that existing data do not allow to analyse the determinants of economic activity, as well as the economic and (wider) social productivity of older people in the desired range. Foregoing applies also to the BKL data sets. At first, despite the broad (in comparison with other surveys, as SHARE, Polish Social Diagnosis, etc.) assessment of competencies in the context of productivity it should include more detailed data. For example, it could be more helpful to break the physical fitness down into subskills: aerobic capacity, strength, flexibility, and balance. With such degree of detail, the recommendations could be more adequate. The second thing is the possibility of productivity measurement (not only in economic, but also in social sense). Most of existing data sets have their own strengths and weaknesses, what makes the drawing conclusions in this area difficult.

## Footnotes

1 Bilans Kapitału Ludzkiego is performed annually in Poland since 2010. At the time of preparation of the analyses, the most up-to-date and accessible public databases (http://bkl.parp.gov.pl/dane) ended on the year 2013. The study is performed as a part of a system project of the Polish Agency for Enterprise Development and the Jagiellonian University, co-financed by the EU.

2 Act from 20 April 2004 on Employment Promotion and Labour Market Institutions **(**Journal of Laws 2004, No. 99, item 1001 as amended).

3 The study used analytical weights levelling the sample structure with the structure of the general population with respect to sex, age, and voivodeship of residence.

4 For a detailed explanation, see Górniak (2011), pp. 34–38.

5 One may attempt to estimate this based on the information about year of birth, job seniority, and year of starting one’s first job. However, this will still be only an estimation due to possible subperiods of economic inactivity which were not reported in the study.

6 Differences evaluated using the Mann–Whitney U test.

7 The selection of variables (from a predeclared set) was made using the stepwise method (Forward Wald, with α = 0.05), whereby non-significant variables and those correlated with other variables are not included in the models. For all variables, the VIF statistic is below 10 in all models (the problem of multicollinearity does not exist).
